# Bovine endometrial stromal cells display osteogenic properties

**DOI:** 10.1186/1477-7827-6-65

**Published:** 2008-12-16

**Authors:** Gaetano Donofrio, Valentina Franceschi, Antonio Capocefalo, Sandro Cavirani, Iain Martin Sheldon

**Affiliations:** 1 Università di Parma, Facoltà di Medicina Veterinaria, Dipartimento di Salute Animale, Sezione di Malattie Infettive degli Animali, Via del Taglio 8, 43100 Parma, Italy; 2 Institute of Life Science, School of Medicine, Swansea University, Singleton Park, Swansea, SA2 8PP, UK

## Abstract

The endometrium is central to mammalian fertility. The endometrial stromal cells are very dynamic, growing and differentiating throughout the estrous cycle and pregnancy. In humans, stromal cells appear to have progenitor or stem cell capabilities and the cells can even differentiate into bone. It is not clear whether bovine endometrial stromal cells exhibit a similar phenotypic plasticity. So, the present study tested the hypothesis that bovine endometrial stromal cells could be differentiated along an osteogenic lineage. Pure populations of bovine stromal cells were isolated from the endometrium. The endometrial stromal cell phenotype was confirmed by morphology, prostaglandin secretion, and susceptibility to viral infection. However, cultivation of the cells in standard endometrial cell culture medium lead to a mesenchymal phenotype similar to that of bovine bone marrow cells. Furthermore, the endometrial stromal cells developed signs of osteogenesis, such as alizarin positive nodules. When the stromal cells were cultured in a specific osteogenic medium the cells rapidly developed the characteristics of mineralized bone. In conclusion, the present study has identified that stromal cells from the bovine endometrium show a capability for phenotype plasticity similar to mesenchymal progenitor cells. These observations pave the way for further investigation of the mechanisms of stroma cell differentiation in the bovine reproductive tract.

## Background

The endometrium is central to normal fertility in all mammals. The endometrium develops in the embryo from the paramesonephric duct, with stromal cells differentiating from uterine mesenchymal cells [1]. The adult endometrium consists of luminal and glandular epithelial cells, stromal cells, vascular smooth muscle cells, endothelial cells, and leukocytes [1]. The stromal cells play a crucial role in directing the development, growth, differentiation, and function of the overlying epithelium in the embryo and adult [2-4]. The stromal and epithelial cells proliferate and differentiate throughout the estrous cycle and during pregnancy, mainly under the control of the ovarian steroid hormones, estradiol and progesterone [5,6]. However, the stromal cells play a critical role as the receptors for estradiol and progesterone are mainly in the stromal rather than the epithelial cells [5-7].

The endometrium is a highly regenerative tissue, and stromal progenitor cells of mesenchymal origin appear to be present in the adult endometrium of at least humans and mice [8]. Normally these progenitor cells differentiate into endometrial stromal cells under the influence of paracrine growth factors and ovarian steroids [7,8]. Functional approaches have been used to identify the candidate endometrial stem/progenitor cells, as there are no specific stem cell markers [8]. For example, the stromal progenitor cells from the human endometrium have the potential to differentiate into other terminal cell lineages including bone, fat and cartilage, in a similar manner to bone marrow-derived mesenchymal cells [8]. It has not been established if stromal progenitor cells exist in the bovine endometrium or whether stromal cells have the capability to differentiate into other lineages.

It is important to explore the plasticity of stromal cells to understand the mechanisms of stromal regeneration and differentiation during reproductive cycles and pregnancy. In addition, stromal cells may differentiate into other lineages in the endometrium that are associated with infertility. Osseous metaplasia of stromal cells is found in the human endometrium and is a sporadic cause of infertility in humans and horses [9,10]; although this has not been investigated in cattle. So, the present study tested the hypothesis that bovine endometrial stromal cells could be differentiated along an osteogenic lineage.

## Methods

### Endometrial cells isolation and primary cultures

Bovine uteri from post-pubertal non-pregnant healthy cross-bred beef animals 18 to 28 months old with no evidence of genital disease were collected at a local abattoir immediately after slaughter for human consumption. The uteri were kept on ice until further processing in the laboratory. The physiological stage of the reproductive cycle for each genital tract was determined by observation of the ovarian morphology [11]. Genital tracts with an ovarian Stage I corpus luteum were selected and the tissue ipsilateral to the corpus luteum was used for endometrial cell isolation by a fractional enzyme dissociation method to avoid potential confounding effects of the stage of estrous cycle or site of tissue collection, as widely established previously [12-15]. The uterine horn ipsilateral to the corpus luteum was opened lengthwise with scissors from the oviduct to the bifurcation of the uterine horns. The endometrium was washed with sterile saline three times and the endometrium carefully dissected free from the underlying tissues with scissors. The endometrium was then cut into strips and placed into serum-free RPMI-1640 (Sigma, Milano, Italy) supplemented with 50 IU/mL of penicillin, 50 μg/mL of streptomycin and 2.5 μg/mL of Amphotericin B (Sigma, Milano, Italy), working under sterile conditions. The strips were then chopped into 1 mm^3 ^pieces, placed into HBSS (Sigma, Milano, Italy) and processed as previously described [15]. Briefly, tissue was digested in 25 mL sterile filtered digestive solution, which was made by dissolving 50 mg trypsin III (Roche), 50 mg collagenase II (Sigma, Milano, Italy), 100 mg Bovine Serum Albumine (BSA; Sigma) and 10 mg DNase I (Sigma) in 100 mL phenol-red free HBSS. Following a 1.5 h incubation in a shaking water bath at 37°C, the cell suspension was filtered through a 40 μm mesh (Fisher Scientific) to remove undigested material and the filtrate was resuspended in a washing medium comprising of phenol-red free HBSS containing 10% Fetal Bovine Serum (FBS) (Sigma) and 3 μg/mL trypsin inhibitor (Sigma). The suspension was centrifuged at 100 × *g *for 10 min and following two further washes in washing medium, the cells were resuspended in RPMI-1640 containing 10% FBS, 50 IU/mL of penicillin, 50 μg/mL of streptomycin and 2.5 μg/mL of Amphotericin B. The cells were plated at a density of 1 × 10^5 ^cells in 2 mL per well using 24-well plates (Nunc). To obtain separate stromal and epithelial cell populations, the cell suspension was removed 18 h after plating, which allowed selective attachment of stromal cells. The removed cell suspension was then replated and incubated allowing epithelial cells to adhere. Stromal and epithelial cell populations were distinguished by cell morphology as previously described [13]. The absence of immune cells in the uterine cell cultures was confirmed by Reverse Transcriptase Polymerase Chain Reaction (RT-PCR) for the Cluster of Differentiation 45 (CD45) pan-leukocyte marker as previously described [15]. The culture media was changed every 48–72 h until the cells reached confluence. All cultures were maintained at 37°C, 5% CO_2 _in air, in a humidified incubator.

### Antibody Array

25 cm^2 ^flasks were seeded with 5 × 10^5 ^of bovine bone marrow stromal cells or bovine endometrial stromal cells in 10 mL of minimal essential medium (EMEM, Lonza, Belgium) containing 10% FBS, 2 mM L-Glutamin 50 IU/mL of penicillin, 50 μg/mL of streptomycin, 2.5 μg/mL of Amphotericin B and maintained at 37°C, 5% CO_2 _in air, in a humidified incubator. 48 h post cells seeding, medium was substituted with 3 mL of serum free medium (EMEM containing, 2 mM L-Glutamin 50 IU/mL of penicillin, 50 μg/mL of streptomycin) and incubated at 37°C, 5% CO_2 _in air, in a humidified incubator. After 24 h the conditioned medium, was removed from the flasks, clarified by high speed centrifugation (14.000 rpm for 5 min at 4°C) and than screened for cytokine secretion using an Antibody Array system as according to the manufacturer's instructions (RayBio, Belgium, Human Cytokine Antibody Array 3.1, RayBiotech, Inc).

### Viral infection

To further characterize the endometrial stromal cells, the peculiar susceptibility to Bovine Herpesvirus-1 (BoHV-4) infection was tested using a recombinant BoHV-4 expressing enhanced green fluorescent protein (EGFP) [16,17]. Recombinant BoHV-4EGFPΔTK was obtained by insertion of the CMV/EGFP expression cassette from the pEGFP-C1 plasmid into the thymidine kynase (TK) locus of the DN 599 BoHV-4 strain [17]. BoHV-4EGFPΔTK and the NADL strain of Bovine Viral diarrhea Virus (BVDV), were propagated by infecting confluent monolayers of Madin-Darby Bovine Kidney cells (MDBK) at a multiplicity of infection (m.o.i.) of 0.5 tissue cell infectious doses/50 (TCID_50_) per cell and maintained in Minimum Essential Medium (MEM) with 2% FBS for 2 h. The medium was then removed and replaced by fresh MEM containing 10% FBS. The virus was purified when approximately 90% of the cell monolayer exhibited a Cytopathic Effect (CPE), at approximately 72 h post-infection (P.I.). Cell-associated virions were freed by three cycles of freezing the flasks at -80°C and thawing. Cell debris was removed by low-speed centrifugation, and virions were pelleted through a 3 ml cushion of 30% sucrose in PBS, in a Beckman 70 Ti rotor at 35,000 rpm for 90 min at 4°C. Viral pellets were resuspended in cold MEM without FBS and TCID_50 _were determined on MDBK cells by limiting dilution [18].

### MTT cell survival assay

The MTT (3-(4,5-dimethylthiazol-2-yl)-2,5-diphenyltetrazolium bromide) cell metabolic assay was used to measure the number of live cells [19]. Briefly, the cell cultures were incubated for 4 h with 100 μg/well MTT before addition of 100 μL of solubilization solution (10% SDS in HCl 0.01 M), and further incubation for 16 h at 37°C. The yellow tetrazolium MTT salt is reduced in metabolically active cells to form insoluble purple formazan crystals, which are solubilized by the addition of a detergent [19]. The optical density was measured at 540 nm, using 690 nm as the reference wavelength in an SLT-Lab microreader (Salzburg, Austria); for each cell type, a linear relationship between cell number and optical density had already been established [20]. Each experiment was repeated three times and each treatment was performed with eight replicates. Statistical differences among treatments were tested by Student's *t *test.

### In vitro osteogenic differentiation

First or second passage bovine endometrial stromal cells were subcultured in six-well plates at a density of 10^6 ^cells per well till confluence and evaluated for their differentiation potential by exposing the cells to osteoinductive medium [21]. Osteogenic differentiation medium consisted of basic medium supplemented with 100 nM dexamethasone, 10 mM-glycerophosphate, and 0.05 mM ascorbic acid-2-phosphate [21]. The cells cultured in basic medium served as controls. On days 3, 7, 14 and 21, cells from both osteoinductive and control groups were washed twice with phosphate-buffered saline and stained [21].

### Staining

At each defined time point, the plated cells were fixed with 10% paraformaldehyde and stained using Alizarin (Sigma) and von Kossa stain (Bio-Optica, Milano, Italy) to evaluate osteogenic differentiation of adipose-derived cells. Extracellular matrix mineral deposition was determined by the presence of black nodules using von Kossa staining, which allows the visualization of calcium deposition by replacing the calcium with silver nitrate [21]. Darker staining of cellular nodules using von Kossa dye reflects more calcium deposition in the extracellular matrix [21]. The level of mineral deposition in the stromal cells stained with von Kossa dye was evaluated by examining 10 randomly selected areas of each culture well using an Axiovert 200 microscope and the associated AxioVision LE Rel. 4.4 densitometry software (Zeiss, Jena, Germany).

### Testing the hypothesis

#### a) Isolation of pure populations of bovine endometrial stromal cells

**Figure 1:**
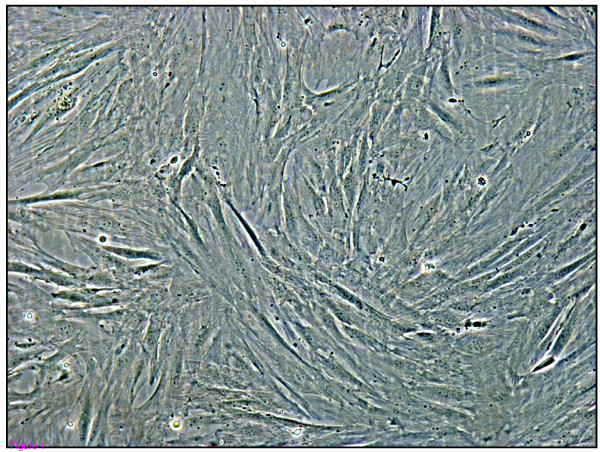
Representative phase contrast images (10×) of pure population of bovine endometrial stromal cells.

**Figure 2:**
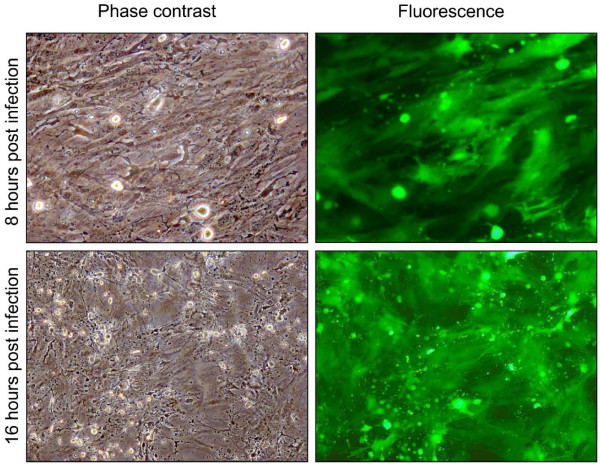
Representative phase contrast images and fluorescence (green) (10×) of bovine endometrial stromal cells at different time (12, 24, 48, 72 h) post infection (P.I.) with 1 m.o.i. of BoHV-4 EGFPΔTK and the respective phase contrast images of uninfected control. Spreading of the infection can be observed by the green colour invading the field during the time and the CPE is morphologically appreciable by the change of the cell shape, where the cells tend to shrink, becoming roundest and detaching the flask surface. The experiment was repeated three times giving the same result. Each experiment was repeated three times giving similar results.

Endometrial cell cultures were established by the fractional enzyme dissociation method described in Materials and Methods, and there was no mRNA expression of the CD45 pan-leukocyte marker in the epithelial or stromal cells (data not shown) as previously described [15]. The stromal cell purity was estimated to be greater than 95% as determined by microscopy (Fig. 1), preferential secretion of prostaglandin E_2 _rather than F_2α_, [12,15] and a high susceptibility to Bovine herpesvirus 4 (Fig. 2) [16].

#### b) Bovine endometrial stromal cells are indistinguishable from bovine bone marrow stromal cells

**Figure 3:**
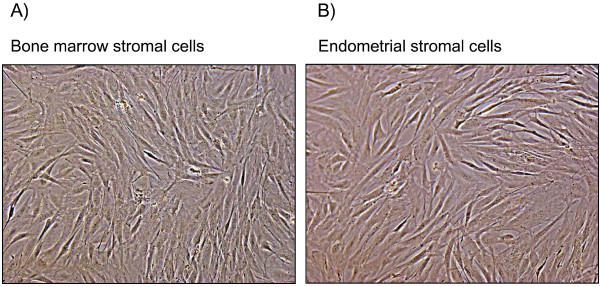
Representative phase contrast images (10×) of pure population of bovine endometrial stromal cells and bovine bone marrow stromal cells with no apparent morphological differences. Each experiment was repeated three times giving similar results.

**Figure 4:**
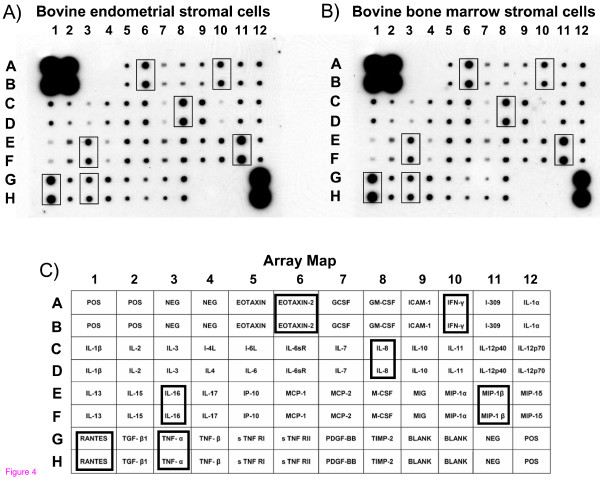
Patterns of secreted molecules into the sovranatant of bovine endometrial stromal cells (a) and bovine bone marrow stromal cells (B), detected by an antibody Array. C) Map of the specific spotted antibodies in duplicate. Positive, negative and BLANK controls are indicated by POS, NEG and BLANK respectively. Squares indicate over expressed and secreted molecules, where RANTES, MIP-1β, EOTAXIN-2, IL-8, IFN-γ, IL-16 and TNF-α are the most abundant in both cellular types. The test was repeated twice, giving identical results.

First or second passage endometrial stromal cells were left to grow until they reached confluence. These cells were morphologically indistinguishable with a confluent monolayer of a first or second passage of bovine bone marrow mesenchymal stromal cells prepared as previously described (Fig. 3) [21]. Such morphological similarity was confirmed in terms of functionality comparing the pattern of constitutively secreted cytokines by an Antibody Array (Fig. 4) [22].

#### c) Long term culture of endometrial stromal cells is accompanied by a morphological change of the cell monolayer

**Figure 5:**
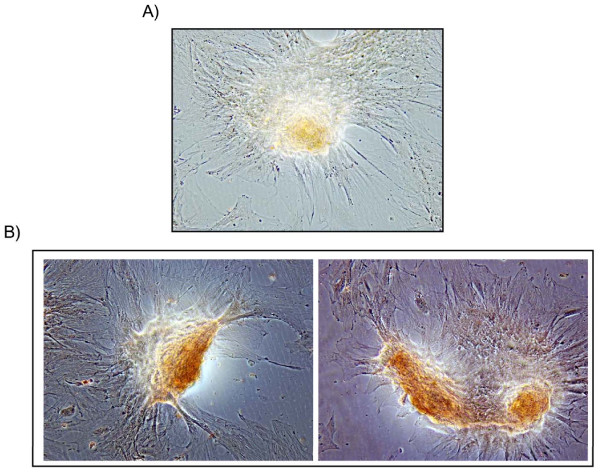
(a) Representative phase contrast images (10×) of bovine endometrial stromal cells long term culture interested by a strong morphological change accompanied by nodules formation, which reacted positively to alizarin staining (b). Each experiment was repeated three times giving similar results.

Confluent monolayers of bovine endometrial stromal cells were left to grow with the medium changed every 48 to 72 h, and cell morphology was examined daily by light microscopy. From the 18^th ^day of each culture a strong morphological change was detectable, where the stromal cells aggregated and formed nodules (Fig. 5a). These morphological changes were not accompanied by cell death as determined by the absence of significant changes in cell numbers during the study period, evaluated using the MTT assay (P = 0.8). The nodules reacted positively to alizarin staining (Fig. 5b), which indicated a possible osteogenic lineage commitment of the bovine endometrial stromal cells.

#### d) Osteogenic medium strongly enhances the osteogenic differentiation of bovine endometrial stromal cells

**Figure 6:**
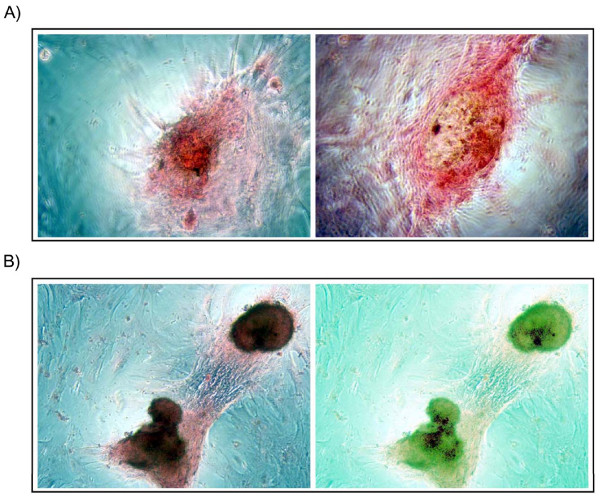
(a) Representative phase contrast images (10×) of bovine endometrial stromal cells at 10 days post induction with osteogenic medium, where nodules formed and started to accumulate extracellular matrix mineral deposition as reflected by the presence of black nodules using von Kossa staining. (b) At 21 days post induction with osteogenic medium nodules increased of number, size, organization as well as mineral deposition. The level of mineral deposition (dark dots) starting from the centre of the nodules (green) in the stromal cells stained with von Kossa dye was evaluated using an Axiovert 200 microscope and the associated AxioVision LE Rel. 4.4 densitometry software (Zeiss, Jena, Germany).

To further test if endometrial stromal cells in long term culture were capable of osteoblastic differentiation, confluent cells were cultured in an osteoinductive medium consisting of growing medium supplemented with h-glycerophosphate, dexamethasone and ascorbic acid [21,23]. When exposed to osteogenic medium, the treated cells changed their morphology within 24 h of treatment and aggregated into nodules, whereas the cells in control medium took 18 days to display the same phenotype. From the second week the treated cells but not the control cells, formed larger nodules enriched with extracellular matrix, which reacted positively to von Kossa staining, indicating the accumulation of calcium (Fig. 6). These data strongly support a bone-type mineralization of the extracellular matrix secreted by differentiated bovine endometrial stromal cells.

### Implications of the hypothesis

Bovine endometrial stromal cells were successfully cultured over several weeks as pure populations without immune cell contamination. When the cultures were established the stromal cells had the expected endometrial phenotype in terms of morphology, secretion of prostaglandin E_2 _and susceptibility to herpesvirus infection. However, in long-term culture the bovine endometrial stromal cells adopted a mesenchymal phenotype similar to bone marrow derived cells, and showed evidence of differentiation along the osteogenic lineage. Furthermore, this bone-like differentiation was enhanced in the presence of osteogenic medium. The implications of the present study are that stromal cells from the bovine endometrium appear to have the plasticity and/or progenitor cell capability to differentiate into bone.

Until a few years ago, commitment to a cell differentiation pathway was believed to be irreversible and it was assumed that cells acquired a terminal phenotype after going through several intermediate steps [24]. Cell lineages were regarded as separate, so that one cell type could not switch to another. However, this was the case in the present study as the endometrial stromal phenotype was able to differentiate into a bone phenotype. This observation supports the more recent concept that cells exhibit phenotypic plasticity [25]. Plasticity is the ability to give rise to cell types whose phenotype is different from that of the source tissue. The environmental cues that direct stromal cell growth and differentiation in the normal endometrium include growth factors and ovarian steroids [5,6,8]. Presumably the absence of epithelial cells, paracrine factors or steroids must allow the stromal cells to differentiate into another lineage.

Non hematopoietic cells of mesodermal derivation exist in a number of postnatal organs and connective tissues, most notably the bone marrow, but also gut, lung, liver, adipose, dental pulp, and periodontal ligament [26,27]. Similar cells have also been isolated from peripheral [28], placental [29], and umbilical cord [30] blood. Typically, these cells occupy a perivascular niche where they support other cell types (e.g., hematopoietic cells) and contribute to the creation and maintenance of different connective and skeletal tissues.

In the present study, stromal cells in osteogenic medium had a more rapid development of bone, which agrees with previous observations that the manipulation of culture conditions can drive various cell types to different lineages including bone [25,31]. Pittenger established that adult marrow stromal cells could differentiate *in vitro *into several cell lines including osteoblasts [25]. However, to induce differentiation into adipocytes, chondrocytes, or osteoblasts, specific factors must be added to the culture medium. Differentiation into osteoblasts, which generates phenotypic characteristics such as alkaline phosphatase and osteopontin production, occurs when cells are exposed to dexamethasone, β-glycerol phosphate, vitamin C, and 10% serum [21]. The present culture system also included serum, but an osteogenic medium was not required to obtain the bone phenotype. However, addition of dexamethasone, glycerophosphate and ascorbic acid certainly enhanced bone formation. Why the stromal cells differentiated along the bone lineage without osteogenic medium is intriguing. Perhaps this reflects their mesenchymal origin [1]. It is also interesting to note that endometrial cells express many genes in common with bone cells, such as those of the Wnt/β-catenin, Transforming Growth Factor-β, Bone Morphogenic Protein, Notch and Hedgehog pathways [32,33]. Furthermore, the endometrial stroma and bone are closely regulated by estradiol, progesterone and insulin-like growth factors [34-36].

Whatever, the signaling pathways involved in differentiating endometrial stromal cells, it does appear that bovine stromal cells can differentiate into bone and this may have clinical relevance. Osseous metaplasia of stromal cells is found in the human endometrium and is a sporadic cause of infertility in humans [9] and horses [10]. Although this has not been investigated in cattle, it has been suggested that acute or chronic inflammation may lead to ossification by metaplasia [37]. Certainly inflammation of the endometrium is extremely common in cattle [38].

In conclusion, the present study has identified that stromal cells from the bovine endometrium show the capability of osteogenic differentiation. The cells in long-term culture undergo spontaneous osteogenic differentiation, and this can be enhanced by specific osteogenic growth factors. These observations pave the way for further investigation of the mechanisms of stroma cell differentiation.

## Competing interests

The authors declare that they have no competing interests.

## Authors' contributions

GD conceived, designed, performed the experiments and wrote the paper. VF and AC contributed to perform the experiments. IMS provided the bovine endometrial stromal cells and contributed to writing of the paper.
